# Association between Serum Osteocalcin and Cardiometabolic Risk Factors in Latent Autoimmune Diabetes in Adults: Insights from Glutamic Acid Decarboxylase Subgroup Analysis

**DOI:** 10.2174/0118715303364381250428100830

**Published:** 2025-05-13

**Authors:** Xiuli Fu, Zihui Xu, Jinlin Xu, Qin Tan, Zhongjing Wang

**Affiliations:** 1 Department of Endocrinology, The Central Hospital of Wuhan, Tongji Medical College, Huazhong University of Science and Technology, Wuhan, China

**Keywords:** Latent autoimmune diabetes in adults, osteocalcin, body mass index, haemoglobin A1c, C-peptide, obesity, cardiometabolic risk

## Abstract

**Aims:**

This study aimed to explore the association between serum osteocalcin and cardiometabolic risk factors in latent autoimmune diabetes in adults (LADA).

**Background:**

Patients with LADA tend to experience poorer glycaemic control, placing them at a higher risk of cardiovascular disease.

**Objective:**

This study aimed to explore the relationship between osteocalcin levels and cardiometabolic risk factors, such as HbA1c, lipids, insulin resistance and obesity, in patients with LADA.

**Methods:**

This retrospective study included 110 patients suffering from LADA with high glutamic acid decarboxylase (GAD) levels (≥180 IU/ml) and 286 with low GAD levels (<180 IU/ml) between January 2018 and December 2021. All participants were aged ≥30 years. Blood samples, collected after 8 hours of fasting, were analysed for osteocalcin and other biomarkers using chemiluminescence immunoassay. Logistic regression analysis assessed the relationship between osteocalcin and cardiometabolic risk factors.

**Results:**

The LADA^high^ group had a lower body mass index (19.5 *vs* 23.4 kg/m^2^, *p* = 0.014) and a lower proportion of obesity (19.1% *vs* 25.2%, *p* = 0.001) compared with the LADA^low^ group. Participants in the LADA^high^ group were slightly older (58.7 *vs* 58.3 years, *p* = 0.641). Fasting blood glucose was higher (8.9 *vs* 8.4 mmol/l, *p* = 0.068), and C-peptide was lower (1.0 *vs* 1.8 ng/ml, *p* = 0.024). Osteocalcin levels were significantly lower in the LADA^high^ group (12.19 *vs* 13.96 ng/ml, *p* = 0.004). Subgroup analyses revealed significant correlations, such as those between osteocalcin and HbA1c in the LADA^low^ group and an inverse relationship with triglyceride in men from the LADA^high^ group. Logistic regression analysis indicated that lower osteocalcin levels were significantly associated with a higher risk of poor glycaemic control and increased obesity.

**Conclusion:**

Lower osteocalcin levels in patients with LADA with high GAD are associated with worse cardiometabolic parameters and increased cardiovascular risk. Osteocalcin may be a potential marker for assessing cardiometabolic risk in patients with LADA.

## 1 INTRODUCTION

Latent autoimmune diabetes in adults (LADA) is a subtype of diabetes with a unique pathophysiology, which includes both autoimmune features and insulin resistance similar to type 2 diabetes (T2DM) [1, 2]. The criteria for defining LADA were as follows: positivity for glutamic acid decarboxylase (GAD) autoantibodies (GADA), age >35 years at diagnosis and no insulin therapy in the first 6–12 months after diagnosis [3]. Studies indicate that the incidence of LADA is higher than classical type 1 diabetes (T1DM), accounting for 4%–14% of patients previously diagnosed with T2DM [4]. Patients with LADA tend to experience poorer glycaemic control due to insufficient insulin secretion and the lack of specific treatment guidelines, placing them at a higher risk of cardiovascular disease (CVD) [5-7]. Notably, cardiovascular outcomes in LADA are comparable with those in T2DM, as demonstrated by the UK Prospective Diabetes Study [8]. A Swedish cohort study on newly diagnosed patients with LADA reported similar rates of CVD and all-cause mortality between LADA and T2DM patients over a 10-year follow-up period [9]. Cardiometabolic risk factors, including insulin resistance, dyslipidaemia and obesity, are thought to underlie the elevated CVD risk in both conditions.

The mechanisms linking LADA to CVDs are complex, involving metabolic dysregulation and autoimmune processes. Chronic hyperglycaemia, insulin resistance and dyslipidaemia contribute to endothelial dysfunction, arterial stiffness and atherosclerosis, accelerating the development of CVD in patients with LADA [10, 11]. Additionally, pro-inflammatory markers, often elevated in autoimmune conditions, such as LADA, may further exacerbate vascular damage, creating a vicious cycle that heightens cardiovascular risk [12].

Osteocalcin, a biomarker of bone formation produced by osteoblasts, has emerged as a potential modulator of insulin sensitivity and energy metabolism [13]. Studies have shown that osteocalcin enhances insulin secretion by stimulating adiponectin and influencing the function of pancreatic β- cells and adipocytes. This affects glucose tolerance, fat consumption and insulin resistance [14-17]. In humans, osteocalcin levels are inversely associated with insulin resistance and fasting glucose levels, suggesting that its role may extend beyond bone metabolism to influence glycaemic control [18] given the higher cardiovascular risk in LADA and its association with metabolic dysregulation, osteocalcin's potential role in modulating cardiometabolic risk warrants further investigation.

Latent autoimmune diabetes in adults is characterised by a slower decline in pancreatic β-cell function than in T1DM, but faster than in T2DM. While the GADA level is considered a sensitive marker for β-cell-specific autoimmunity, its predictive value for β-cell exhaustion remains debated. Some studies highlight its predictive accuracy, while others report variability in its association with β-cell function [19-22]. Latent autoimmune diabetes in adults is further divided into low- and high-titre subtypes according to the GADA level, with low-titre LADA resembling T2DM and high-titre LADA showing clinical similarities to T1DM, including a more rapid decline in islet function [23, 24].

Although osteocalcin has been linked to cardiovascular and metabolic risk factors in T2DM, few studies have examined its role in LADA. Therefore, this study aims to explore the relationship between osteocalcin levels and cardiometabolic risk factors, such as HbA1c, lipids, insulin resistance, and obesity, in patients with LADA, providing new insights into potential mechanisms linking LADA and cardiovascular risk.

## 2 METHODS

### 2.1 Participants

Patients with LADA were enrolled at The Central Hospital of Wuhan between January 2018 and December 2021. The inclusion criteria were as follows: patients diagnosed with LADA, aged ≥30 years at diagnosis, GADA-positive, and insulin-independent (insulin use <1 month) for 6 months after diagnosis [25, 26]. The exclusion criteria were as follows: (1) acute complications, such as ketoacidosis and hyperosmolar coma; (2) patients suffering from metabolic bone diseases or bone tumours; (3) incomplete clinical data. The clinical data were extracted from the electronic medical record system, including the patient's gender, age, height, weight, systolic and diastolic blood pressure, and the duration of diabetes. Body mass index (BMI) was defined as weight (kg)/height^2^ (m^2^). This study was approved by the Human Ethics Committee of Wuhan Central Hospital with the approval number WHZXKYL2022-073 and was conducted in accordance with the Declaration of Helsinki. As our study was retrospective, the Human Ethics Committee of Wuhan Central Hospital waived the requirement of informed consent.

### 2.2 Clinical Parameters and Data Collection

Fasting blood samples were collected from the antecubital vein after 8 hours of fasting, and biomedical measurements were taken. Blood samples were used to measure serum uric acid (SUA), C-reactive protein, HbA1c, fasting blood glucose (FBG), serum high-density lipoprotein cholesterol (HDL), triglyceride (TG), low-density lipoprotein cholesterol (LDL), serum total cholesterol (TC) and liver and kidney function. Serum C-peptide and osteocalcin were assessed by chemiluminescence immunoassay (Roche Diagnostics Ltd., Basel, Switzerland) and measured on the Roche Cobas e411 analyser. The usual biochemical results were obtained using standard clinical practice methods, where kits from mixed batches were used, reflecting real-world conditions. The Homeostatic Model Assessment for Insulin Resistance (HOMA-IR) was calculated using a HOMA calculator based on fasting C-peptide and glucose levels [27]. Hypertension was defined as high blood pressure with a systolic blood pressure ≥140 mmHg and/or a diastolic blood pressure ≥90 mmHg after repeated tests or a doctor's diagnosis. The glomerular filtration rate was estimated using the collaborative formula of chronic kidney disease epidemiology. The BMI was calculated by dividing the patient's weight in kilograms by the square of their height in metres.

### 2.3 Cardiometabolic Risk Factors

High TC was considered to be ≥4.5 mmol/l; high TG was considered to be ≥1.7 mmol/l; high LDL-C was considered to be ≥2.6 mmol/l; low HDL-C was considered to be <1.3 mmol/l in women or <1.0 mmol/l in men; hyperuricaemia was considered to be SUA ≥420 μmol/l; poor blood glucose control was considered as HbA1c ≥7% or FBG >7.0 mmol/l. These definitions are based on established clinical guidelines [28]. According to the Chinese population-specific guidelines, 24 < BMI < 28 kg/m^2^ is considered overweight and a BMI ≥28kg/m^2^ is considered obese [29].

### 2.4 Statistical Analysis

The SPSS 26.0 software package (IBM Statistics Group) was used for all analyses. Clinical features of patients were described using the percentage of categorical variables and the mean and standard deviation of continuous variables. The Student *t*-test or one-way analysis of variance was used to explore normally distributed data, and the Bonferroni correction was used for post hoc analysis. Data with a non-normal distribution were analysed by the Kruskal–Wallis H test or Mann–Whitney test to determine the difference between groups. Categorical variables describe the percentage and amount of each type, using chi-squared or Fisher precision tests for comparison between groups. Spearman rank correlation for non-parametric variables and Pearson correlation for parametric variables were used to evaluate the relationship between clinical biomarkers and osteocalcin. After adjusting for various confounders, logistic regression analyses were applied to determine the correlation between cardiometabolic risk factors and osteocalcin levels. A *p*-value of <0.05 was considered a statistically significant difference.

Power analysis was performed using PASS software (NCSS LLC) to determine the required sample size for logistic regression analysis. The analysis aimed to detect differences in cardiometabolic risk factors associated with osteocalcin levels between the high- and low-GAD groups. The effect size was estimated based on the odds ratios (ORs) from the data, with high HbA1c showing an OR of 0.819 in women with high GAD and an OR of 0.942 in women with low GAD. A significance level (alpha) of 0.05 and a desired power of 80% was set. Based on these parameters, the calculation indicated that a minimum of 150 participants per group was needed to achieve sufficient power for detecting significant differences. This ensured that the study was adequately powered to identify meaningful associations between cardiometabolic risk factors and osteocalcin levels.

## 3 RESULTS

Anthropometric and biochemical variables associated with glucose homeostasis and cardiometabolic risk are shown in Table **1**. A total of 396 patients with LADA were included in this study, of whom 52.8% were female and 47.2% were male. Patients were divided into the LADA^low^ group (GAD <180 IU/ml) and the LADA^high^ group (GAD ≥180 IU/ml) according to GAD titres. Comparisons of laboratory measurements and demographic data between groups are summarised in Table **1**. The average BMI of the LADA^low^ group was higher than that of the LADA^high^ group (23.4 ± 5.1 kg/m^2^ *vs* 19.5 ± 4.6 kg/m^2^, *p* = 0.014). The incidence of obesity in the LADA^low^ group was higher than that in the LADA^high^ group (25.2% *vs* 19.1%, *p* < 0.001). Serum levels of HbA1c in the LADA^high^ group were higher than those in the LADA^low^ group. Serum TG levels, C-peptide and osteocalcin levels in the LADA^low^ group were higher than those in the LADA^high^ group (Table **1**).

To explore the correlation between osteocalcin levels and other variables, correlation analyses were performed for each of the four groups. The study showed that osteocalcin was inversely related to BMI in men, whereas this correlation was not evident in women. Osteocalcin levels were significantly correlated with HbA1c in all groups. Osteocalcin levels were closely correlated with FBG except for women in the LADA^low^ group. Osteocalcin was inversely related to high TG risk among the LADA^high^ group in men. There was a positive correlation between osteocalcin level and C-peptide level in female patients and the LADA^high^ group (*r* = 0.124, *p* = 0.01). There was no relationship between HOMA-IR and osteocalcin levels among all groups (Table **2**).

Logistic regression analysis was performed to determine the independent association of serum osteocalcin concentrations with cardiometabolic risk factors. After adjusting for clinical and biochemical variables, including history of CVD, duration of diabetes, smoking status and age, osteocalcin levels seemed to besignificantly related to the risk of obesity and overweight with the LADA^low^ group in men. Serum osteocalcin levels were found to be significantly related to the risk of high HbA1c in all groups. Except for women in the LADA^low^ group, osteocalcin levels were strongly associated with the risk of fasting hyperglycaemia. Osteocalcin levels were related to TG with the LADA^high^ group in men, but not with the risk of high LDL, high TC and low HDL-C (Table **3**).

## 4 DISCUSSION

The stratification by GAD levels and gender provided valuable insights into subgroup-specific relationships between osteocalcin and metabolic risk factors. Unlike previous studies that primarily examined osteocalcin in T2DM, our research highlights the specific metabolic implications of osteocalcin in LADA. Further, it distinguishes its impact between high-titer (LADA^high^) and low-titer (LADA^low^) GAD subgroups. GAD levels are a hallmark of β-cell autoimmunity and a key marker for identifying heterogeneity within LADA [30]. In this cross-sectional study of 396 patients with LADA, osteocalcin levels in the LADA^low^ group were significantly higher than those in the LADA^high^ group. Osteocalcin levels were notably correlated with the risk of high HbA1c across all groups, emphasising its potential role in glucose regulation. Specifically, serum osteocalcin levels were positively associated with C-peptide levels in the LADA^high^ group, indicating a possible link between osteocalcin and endogenous insulin production. This association underscores osteocalcin's potential as a marker of bone turnover and its influence on glucose metabolism; this finding aligns with recent studies suggesting osteocalcin's role in glucose regulation through pancreatic cell proliferation and insulin secretion [31-33].

Despite the theoretical basis for osteocalcin's role in glucose metabolism, clinical evidence remains mixed. Some studies have reported no significant role of osteocalcin in glucose metabolism among patients with T1DM [34, 35], while others have demonstrated a significant association with T2DM [25, 36]. Our study supports these findings by showing a correlation between osteocalcin and HbA1c, which was particularly significant in the LADA^low^ group. This observation may reflect better-preserved β-cell function in this subgroup, which enhances osteocalcin’s metabolic effects on glucose regulation. However, we observed no relationship between osteocalcin and HOMA-IR in our LADA population. This is consistent with studies in patients with T2DM, which also showed variable associations [37, 38]. One possible explanation is that osteocalcin primarily influences insulin secretion rather than insulin sensitivity, particularly in conditions where β-cell dysfunction is not severe. This hypothesis is further supported by our finding that osteocalcin levels correlated positively with C-peptide levels in the LADA^high^ group, suggesting a potential link between osteocalcin and residual β-cell function. Notably, in Japanese men aged >65 years, osteocalcin was inversely related to the glycaemic index and insulin resistance [39], suggesting that age and demographic factors may influence these relationships. Given the lack of consensus in existing literature, further investigations are needed to clarify osteocalcin’s precise role in glucose metabolism among different diabetes subtypes. Future longitudinal studies should assess whether osteocalcin levels predict glycaemic deterioration in LADA and whether interventions targeting osteocalcin pathways, such as vitamin K supplementation or exercise therapy, could improve glucose control. Moreover, mechanistic studies are warranted to explore whether osteocalcin’s effects on glucose homeostasis are mediated through direct pancreatic β-cell modulation, osteoblast-derived hormonal pathways, or interactions with inflammatory processes.

Our results also revealed that osteocalcin levels were inversely associated with BMI in men but not in women. The complex interplay between body fat and bone tissue, which can vary by gender and age, may contribute to this observation [40]. Increased BMI often correlates with higher bone mineral density, yet obesity can alter bone regulatory hormones and reduce bone mass. Previous studies have similarly shown an inverse correlation between osteocalcin levels and BMI, particularly in obese or overweight individuals [41, 42]. However, inconsistent findings regarding this relationship in T2DM patients highlight the need for further research.

Regarding lipid metabolism, our study found that osteocalcin levels were inversely related to high TG risk among men in the LADA^high^ group, which may point to gender-specific variations in lipid metabolism influenced by osteocalcin. This contrasts with studies indicating no significant relationship between osteocalcin and lipid profiles in T2DM [43, 44]. The role of osteocalcin in lipid metabolism remains unclear, but recent research suggests it may influence lipid metabolism through adipocyte-related pathways [45, 46].

Osteocalcin's potential bidirectional regulation of cardiometabolic risk factors and its connection to CVD warrant further investigation. Osteocalcin's association with adverse cardiovascular events and its endothelial protective role in atherosclerosis highlight its importance in cardiovascular health [47-50]. The bidirectional nature of osteocalcin's regulation of cardiometabolic risk factors necessitates a deeper exploration of underlying mechanisms.

This study is novel in its comprehensive exploration of the relationship between cardiometabolic risk factors and osteocalcin levels in the LADA population. Our findings contribute to the growing body of evidence that osteocalcin is associated with multiple cardiovascular risk factors, including high HbA1c, high FBG, high TG and overweight/obesity. The observed sex differences in bone turnover rates highlight the need for gender-specific considerations in clinical practice.

Clinically, these findings suggest that osteocalcin could serve as a useful biomarker for identifying individuals at higher risk of cardiovascular complications in patients with LADA. Integrating osteocalcin measurements into routine clinical assessments could improve risk stratification and personalised management strategies. For example, patients with low osteocalcin levels may benefit from more intensive monitoring and interventions aimed at controlling glycaemic levels and managing weight and lipid profiles. Furthermore, understanding the role of osteocalcin in bone metabolism and its relationship with cardiometabolic risk factors could guide future therapeutic approaches and preventive measures in this patient population.

However, there are limitations to our study. First, the cross-sectional design prevents us from establishing causal relationships between osteocalcin and clinical outcomes. Second, as the study focused on hospitalised patients with LADA, the generalisability of the results is limited. Additionally, since osteocalcin levels can fluctuate over time, the study's findings reflect the state at the time of admission. Last, stratifying by GAD levels and gender provides valuable insights into subgroup-specific relationships between osteocalcin and cardiometabolic risk factors; however, a broader multivariate model adjusting for key covariates would complement these findings and provide a more holistic understanding.

## CONCLUSION

Serum osteocalcin levels in patients with LADA were significantly correlated with cardiometabolic risk factors. Even after accounting for covariates, reduced osteocalcin levels lead to more serious cardiometabolic risks, including obesity, hyperglycaemia and dyslipidaemia.

## Figures and Tables

**Table 1:** Clinical characteristics of total subjects according to GAD values.

-	LADA ^high^	LADA ^low^	*p*-value
GAD(IU/ml)	≥180	<180	-
N	110	286	-
Male(%)	53(48.2%)	134(46.9%)	0.148
Ages, years	58.7±15.3	58.25±16.5	0.641
Duration, years	6.6±2.3	6.95±3.1	0.125
Smoking, (%)	29.1%	30.8%	0.095
Drinking, (%)	26.4%	28.0%	0.128
Hypertension, (%)	27.3%	30%	0.062
CVD history, (%)	16.4%	18.9%	0.075
BMI, kg/m^2^	19.5±4.6	23.4±5.1	0.014
Obesity, n(%)	21(19.1%)	72(25.2%)	0.001
CRP	4.28±2.97	4.09±3.02	0.415
SBP, mmHg	126.9±17.8	128.4±18.4	0.219
DBP, mmHg	70.4±17.3	71.9±18.1	0.256
HbA1c	9.5±2.8	9.1±2.3	0.039
TC, mmol/L	4.2±0.9	4.5±1.0	0.089
TG, mmol/L	1.4±0.8	1.6±0.7	0.025
HDL-C, mmol/L	1.0±0.5	1.1±0.4	0.098
LDL-C, mmol/L	2.5±0.7	2.7±0.8	0.052
ALT	20.0±12.8	21.8±14.1	0.409
AST	20.9±13.1	20.6±13.7	0.382
UA	324.8±144.2	325.7±147.6	0.479
Cr	64.8±30.9	63.7±31.7	0.176
eGFR, ml/min/1.73^2^	81.3±12.5	80.6±13.9	0.275
FBG, mmol/L	8.9±2.8	8.4±3.1	0.068
C peptide	1,0±0.8	1.8±0.7	0.024
HOMA-IR	1.6±0.7	1.7±0.7	0.089
Serum osteocalcin	12.19	13.96	0.004

**Note:** Values are expressed as median (interquartile range), or number (%) of participants. A Kruskal-Wallis test, or chi-square test was used to compare each parameter between groups. **Abbreviations:** GAD: glutamic acid decarboxylase, CVD: cardiovascular disease,BMI:body mass index, CRP: c-reactive protein, HbA1c: Glycosylated Hemoglobin, HDL: high density lipoprotein, LDL: low density lipoprotein, TG: triglycerides, TC: total cholesterol, ALT: alanine aminotransferase, AST: aspartate aminotransferase, UA: uric acid, eGFR: estimated glomerular filtration rate, FBG: fasting blood glucose, HOMA-IR: homeostasis model assessment of insulin resistance. LADA: latent autoimmune diabetes in adults.

**Table 2:** Correlation of serum osteocalcin levels with other variables according to GAD values and sex.

-	LADA ^high^				LADA ^low^			
-	Women		Men		Women		Men	
	r	P	r	P	r	P	r	P
Ages, years	0.105	0.235	0.098	0.342	0.117	0.219	0.101	0.306
BMI, kg/m^2^	-0.135	0.075	-0.189	0.002	-0.107	0.112	-0.196	0.001
CRP	-0.037	0.581	0.064	0.326	-0.082	0.269	-0.029	0.614
SBP, mmHg	0.062	0.329	0.053	0.417	0.068	0.296	-0.046	0.487
DBP, mmHg	-0.052	0.471	0.041	0.523	0.059	0.368	0.069	0.294
HbA1c	-0.329	<0.001	-0.198	<0.001	-0.118	0.01	-0.216	<0.001
TC, mmol/L	0.176	0.098	0.107	0.159	-0.084	0.217	0.132	0.112
TG, mmol/L	0.096	0.105	-0.168	0.037	0.109	0.097	-0.137	0.068
HDL-C, mmol/L	0.067	0.317	0.073	0.296	-0.049	0.487	0.042	0.519
LDL-C, mmol/L	0.093	0.109	0.051	0.436	-0.082	0.216	0.039	0.598
UA	0.058	0.317	-0.073	0.259	0.039	0.481	-0.031	0.522
Cr	0.097	0.183	0.069	0.276	0.125	0.112	0.093	0.198
eGFR, ml/min/1.73^2^	-0.089	0.193	0.064	0.289	0.049	0.347	0.041	0.402
FBG, mmol/L	-0.278	<0.001	-0.241	<0.001	-0.072	0.206	-0.106	0.03
C peptide	0.124	0.01	0.079	0.143	0.052	0.279	0.081	0.107
HOMA-IR	-0.141	0,198	0.129	0.251	0.107	0.307	0.135	0.239

**Note:** Spearman rank-order correlation was used to determine the strength of the relationships. **Abbreviations:** BMI: body mass index, CRP: c-reactive protein, HbA1c: Glycosylated Hemoglobin, HDL: high density lipoprotein, LDL: low density lipoprotein, TG: triglycerides, TC: total cholesterol, ALT: alanine aminotransferase, AST: aspartate aminotransferase, UA: uric acid, eGFR: estimated glomerular filtration rate, FBG: fasting blood glucose, HOMA-IR: homeostasis model assessment of insulin resistance. LADA: latent autoimmune diabetes in adults.

**Table 3:** Relationship between cardiometabolic risk factors and osteocalcin levels according to GAD values and sex.

-	LADA ^high^				LADA ^low^			
-	Women		Men		Women		Men	
-	OR(95%CI)	P	OR(95%CI)	P	OR(95%CI)	P	OR(95%CI)	P
Overweight/Obesity	0.981(0.924,1.034)	0.217	0.965(0.941,1.002)	0.063	0.972(0.956,1.021)	0.135	0.921(0.892,0.976)	0.01
High HbA1c	0.819(0.713,0.914)	<0.001	0.897(0.836,0.975)	<0.001	0.942(0.824,0.982)	0.018	0.872(0.842,0.939)	<0.001
High FBG	0.873(0.809,0.943)	<0.001	0.944(0.872,0.981)	<0.001	0.921(0.845,1.043)	0.098	0.893(0.824,0.961)	<0.001
Hypertension	1.021(0.896-1.194)	0.396	1.037(0.884,1.159)	0.512	0.943(0.812-1.109)	0.221	1.034(0.852,1.298)	0.145
High UA	1.039(0.904,1.156)	0.217	1.041(0.889-1.237)	0.443	1.008(0.873,1.197)	0.298	0.916(0.802-1.104)	0.547
High TC	1.014(0.879-1.112)	0.583	1.036(0.894-1.125)	0.301	0.954(0.882,1.248)	0.298	1.028(0.824,1.214)	0.449
High TG	1.029(0.782-1.297)	0.357	0.907(0.812,0.953)	<0.001	1.041(0.812-1.187)	0.413	1.033(0.913,1.195)	0.576
High LDL-c	0.942(0.817-1.399)	0.592	1.048(0.845-1.298)	0.479	0.923(0.809-1.113)	0.422	1.039(0.876-1.229)	0.298
Low HDL-c	1.034(0.822-1.284)	0.285	1.069(0.932-1.391)	0.581	1.047(0.913-1.116)	0.439	0.983(0.891-1.124)	0.511

**Abbreviations:** LADA: latent autoimmune diabetes in adults; GAD: glutamic acid decarboxylase, FBG: fasting blood glucose, HbA1c: Glycosylated Hemoglobin, HDL: high density lipoprotein, LDL: low density lipoprotein, TG: triglycerides, TC: total cholesterol, UA: uric acid.

## Data Availability

All data generated or analysed during this study are included in this article. Further inquiries can be directed to the corresponding author.

## LIST OF ABBREVIATIONS

LADA: Latent Autoimmune Diabetes Adults
GAD: Glutamic Acid Decarboxylase
T2DM: Type 2 Diabetes
T1DM: Type 1 Diabetes

## STANDARDS OF REPORTING

STROBE guidelines were followed.
